# Altered Regional Gray Matter Volume in Obese Men: A Structural MRI Study

**DOI:** 10.3389/fpsyg.2017.00125

**Published:** 2017-01-31

**Authors:** Bin Zhang, Xiao Tian, Derun Tian, Jinhong Wang, Qiming Wang, Chunshui Yu, Chunbo Li, Jijun Wang

**Affiliations:** ^1^Shanghai Key Laboratory of Psychotic Disorders, Shanghai Mental Health Center, Shanghai Jiao Tong University School of MedicineShanghai, China; ^2^Key Laboratory of Cancer Immunology and Biotherapy, Biotherapy Center, Tianjin Medical University Cancer Institute and Hospital, National Clinical Research Center of CancerTianjin, China; ^3^Department of Anatomy, Tianjin Medical UniversityTianjin, China; ^4^Department of Medical Imaging, Shanghai Mental Health Center, Shanghai Jiao Tong University School of MedicineShanghai, China; ^5^Department of Radiology, Tianjin Medical University General HospitalTianjin, China

**Keywords:** obesity, functional MRI, gray matter volume, hunger rating, insulin, putamen

## Abstract

Obesity is associated with a number of health problems, especial insulin resistance and Type 2 diabetes. Our previous study showed that obese males had decreased neural activity in the orbital frontal cortex (OFC) and increased activity in the left putamen (Zhang et al., 2015b), which could indicate altered eating behaviors in obesity related to a hyper-functioning striatum and hypo-functioning inhibitory control. Accordingly, our goal of the current study was to determine whether there are alterations in the brain structures within these two neural systems in obese individuals. Twenty obese men (age: 20–28 years) and 20 age-matched lean male subjects were involved in the current study. Plasma glucose and insulin were tested during hunger state, and homeostasis model assessment of insulin resistance (HOMA-IR) was based on the blood samples. In the study, we used structural MRI and a voxel-based morphometry (VBM) method to investigate regional structures in obese subjects and find out whether there are correlations between the insulin and the brain structures. We found that obese men only showed a significantly increased gray matter volume (GMV) in the left putamen and that the GMV of the left putamen was positively correlated with body mass index, plasma insulin and HOMA-IR. The putamen is a core region participating in insulin signal regulation, and our results showed an abnormal GMV of the putamen is a core alternation in aberrant insulin. Furthermore, the GMV of the OFC was negatively correlated with hunger rating, despite there being no significant difference between the two groups in the OFC. In conclusion, the altered structure and function of the putamen could play important roles in obesity and aberrant insulin.

## Introduction

Obesity is a public health challenge worldwide, and a number of health problems are related to it (Flegal et al., 2007; Renehan et al., 2008). In particular, obesity is linked to insulin resistance, which is the major factor leading to Type 2 diabetes (Prince et al., 2014). The factors causing obesity are multiple, and its etiology is not well understood. However, excessive weight gain is largely due to energy imbalances in calories consumed over calories expended (Janssen et al., 2005; Stice et al., 2006), so abnormal eating behavior has become a core factor in clarifying the obesity epidemic. The hypothesis of altered eating behaviors in obesity is related to three neural systems: a hyper-functioning striatum, hypo-functioning inhibitory control and altered insula (He et al., 2014a,b). However, there is no study investigating whether the structure of these systems altered in obese individuals.

Functional imaging techniques have been applied to investigate behaviors in obesity, and the functional MRI studies have found that dysregulation of eating behaviors serves as a pivotal pathophysiologic feature in obesity (Passamonti et al., 2009; Kullmann et al., 2013b; He et al., 2014a,b; Tuulari et al., 2015). Tuulari et al. (2015) found that obese subjects had lower responses in the medial prefrontal cortex (PFC), a core region of inhibitory control, viewing the stimuli passively or imagining eating the foods. Passamonti et al. (2009) found that the viewing of appetizing foods compared with bland foods produced changes in the appetitive network, suggesting that the findings might determine an individual’s risk of obesity. In our previous study, we used resting state functional MRI method to observe spontaneous neural activity during hunger state in obese individuals and found that obese males had decreased activity in the orbital frontal cortex (OFC) and increased activity in the left putamen (Zhang et al., 2015b), which is consistent with the altered eating behaviors hypothesis (He et al., 2014a,b).

Previous MRI studies have also shown that regional structures are altered in obese individuals (Pannacciulli et al., 2006; Brooks et al., 2013; Lou et al., 2014; He et al., 2015). One study showed that gray matter atrophy in obese subjects occurred in the left PFC, bilateral cingulate cortex and bilateral putamen (Lou et al., 2014). Another study found that gray matter volumes (GMVs) in obese participants were smaller in the bilateral supplementary motor area, left inferior frontal gyrus and left postcentral gyrus (Brooks et al., 2013). However, Sharkey et al. (2015) did not find any significant association between cortical thickness and body mass index (BMI) in children. Due to the inconsistent findings of previous studies, in the current study, we used voxel-based morphometry (VBM) methods to investigate whether there are altered regional structures in obese subjects, especially in striatum and impulse control systems.

Insulin plays an important role in regulation of food intake (Zhao and Alkon, 2001). Previous studies have shown that insulin signals could modify the neural circuitry and regulate whole body energy homeostasis (Figlewicz and Sipols, 2010). Kullmann et al. (2013a) found that intranasal insulin application induced increased activation in hypothalamus. Furthermore, our previous functional MRI study showed that the negative correlation between plasma insulin and the regional brain activity (Zhang et al., 2015a). However, there is no study investigating the relationship between the GMV of the region related to obesity and plasma insulin.

In the present study, we used VBM methods to investigate whether there are altered regional structures in obese subjects. The purpose of the study was to find out the altered structures in obese subjects and the relationship between the GMV of the altered brain region and plasma insulin level. The investigation was motivated by two hypotheses: (1) regional structures of the striatum and impulse control systems are altered in obese individuals, and (2) there is the positive correlation of the altered regional GMV with plasma insulin.

## Materials and Methods

### Subjects

Twenty obese men (age: 20–28 years) and 20 age-matched lean male subjects (age: 20–28 years) were recruited via print poster advertisement in university campuses of Tianjin. Lean subjects were required to have a BMI from 18.5 to 23.9 kg/m^2^, and obese subjects were required to have a BMI > 28 kg/m^2^ using the adjusted Chinese guideline, which is an equivalent of WHO class I obesity (He et al., 2007). None of the subjects had a history of illicit drug dependence or alcohol abuse, and they were not currently dieting to lose weight. Exclusion criteria also included psychiatric medical illnesses, history of seizures, and pregnancy. This study was performed in accordance with the guidelines of the International Committee of Medical Journal Editors. This study was approved by the institutional review board of Tianjin Medical University General Hospital. All of the participants provided written informed consent in accordance with the Helsinki declaration.

### Procedure

All of the subjects completed the paradigm between 5:30 PM and 8:00 PM. On the day of the scan, the subjects fasted for 6–8 h prior to scanning. After lunch, the subjects were asked not to ingest anything except for water until the beginning of the experiment. We assessed the hunger rating using visual analog scales, in which the subjects were asked to rate their sensations of hunger from 0 (‘not at all hungry’) to 100 (‘very hungry’) at the moment. Each subject should mark somewhere suited their sensations of hunger on the visual analog scale. At the beginning of the study, first seven subjects were not taken this test. Then we calculated the score according to their markers. After the assessment of sensations of hunger, all subjects immediately went to the scanning room to do the MRI test.

### Blood Samples Acquisition

Blood samples were obtained from the cubital vein before the scan session. Plasma glucose concentrations were determined by an automated clinical chemistry analyzer (Medical Cooperation, USA), and plasma insulin concentrations were determined by chemiluminescence immunoassay (Siemens Diagnostics, USA). Based on the blood samples, homeostasis model assessment of insulin resistance (HOMA-IR) was calculated as HOMA-IR = glucose (mmol/L) × insulin (mU/L/) 22.5. HOMA-IR is a method used to quantify insulin resistance, the higher value of HOMA-IR, higher level of insulin resistance (Matthews et al., 1985).

### Image Data Acquisition

Brain imaging data were acquired with a 3T MR imaging system (Signa-HDx, General Electric, USA). The subjects’ heads were fixed using foam pads to minimize head motion, and earplugs were used to reduce the scanning noise. Structural images were acquired using a 3D magnetization-prepared rapid-acquisition gradient echo sequence with the following parameters: repetition time = 2000 ms, echo time = 2.6 ms, inversion time = 900 ms, flip angle = 9°, matrix = 256 × 224, field of view = 256 mm × 224 mm, and 176 continuous sagittal slices with a 1 mm thickness. The structural scan time is 352 s.

### Voxel-Based Morphometry Procedure

Structural images were processed using Statistical Parametric Mapping software (SPM8^1^). Images were transformed using Voxel Based Morphometry Toolbox (VBM8), which includes segmentation, bias correction, and normalization using diffeomorphic anatomical registration and the exponentiated lie algebra technique (Ashburner, 2007) with a pre-defined tissue probability map registered to the Montreal Neurological Institute space. Modulation was performed to compensate for the effects of non-linear transformations. Finally, a Gaussian filter of 8 mm full width at half maximum was applied to increase the signal-to-noise ratio.

### Statistical Analyses

We used the independent samples *t*-test to compare group differences in plasma glucose, plasma insulin, HOMA-IR and hunger rating (in subjects with obesity and lean male subjects).

The differences between obese subjects and healthy controls (HCs) were examined with the independent samples *t*-tests between the two groups to create a group difference map. Threshold correction was undertaken by family-wise error (FWE) using SPM with the threshold at a voxel *p* value of *p* < 0.05.

For OFC as a core region of hypo-functioning inhibitory control in obese individuals with *a priori* hypothesis (Zhang et al., 2015b), the search for GMV changes within the OFC was confined accordingly by performing a small volume correction based on the results of our previous study (Zhang et al., 2015b), extracting the OFC (one different activity region between two groups) as a mask. To account for multiple comparisons within this considerably smaller volume of interest, we applied FWE.

For insula as a core region regulating in eating behaviors (He et al., 2014a,b), the search for GMV changes within bilateral insula was confined by performing a small volume correction, extracting the bilateral insula as a mask using WFU PickAtlas^2^. To account for multiple comparisons within this considerably smaller volume of interest, we applied FWE.

We then selected regions of interest from the VBM results (left putamen) and our previous study (OFC). Then, we entered the GMV of the left putamen into correlation analyses with BMI, HOMA-IR and plasma insulin, and we entered the GMV of the OFC into correlation analyses with subjective hunger ratings.

## Results

Plasma glucose levels were similar between the lean and obese subjects (*p* > 0.05). The plasma insulin concentrations and HOMA-IR of obese individuals were significantly higher (*p* < 0.05) than those of lean subjects. However, there were no differences in hunger ratings between the groups (*p* > 0.05; **Table 1**).

**Table 1:** Characteristics of the study population.

	Lean (*n* = 20)	Obese (*n* = 20)	Group effect *P* value
Age (y)	20∼28	20∼28	
Body weight (kg)	63.52 ± 5.66	100.51 ± 13.32	0.015
BMI (kg/m^2^)	21.48 ± 1.43 (range: 18.5–23.9)	33.56 ± 3.53 (range: 28.0–41.5)	0.004
Glucose (mmol/L)	4.46 ± 0.44	4.12 ± 0.72	0.142
Insulin (uU/mL)	4.84 ± 5.30	14.81 ± 11.32	0.001
Hunger Ratings (mm)	71.56 ± 9.61	72.92 ± 13.24	0.330
HOMA-IR	0.80 ± 0.47	2.87 ± 2.96	0.006

**FIGURE 1:**
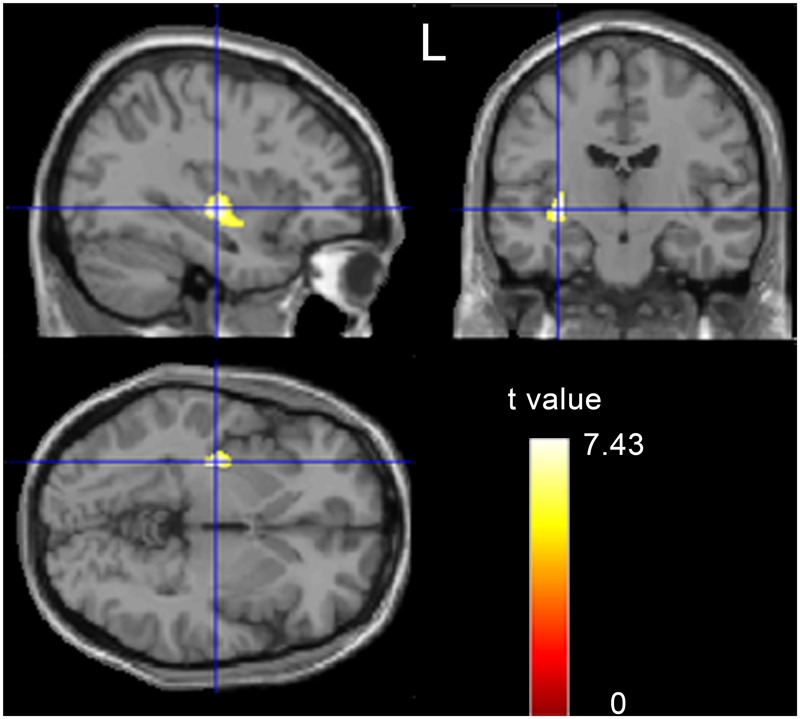
**A t-statistic map showed the gray matter volume (GMV) differences between obese subjects and controls (*p* < 0.05, corrected)**.

On the whole brain level, VBM analysis revealed that obese men showed a significantly increased GMV in the left putamen (*x* = -33, *y* = 63, *z* = -9, *k* = 331, *T* = 7.43, *p* < 0.05, FWE correction; **Figure 1**). No other brain regions showed significant GMV changes.

Within the OFC, the small volume correction results did not reveal any regions with significant differences in GMV between controls and obese men.

**FIGURE 2:**
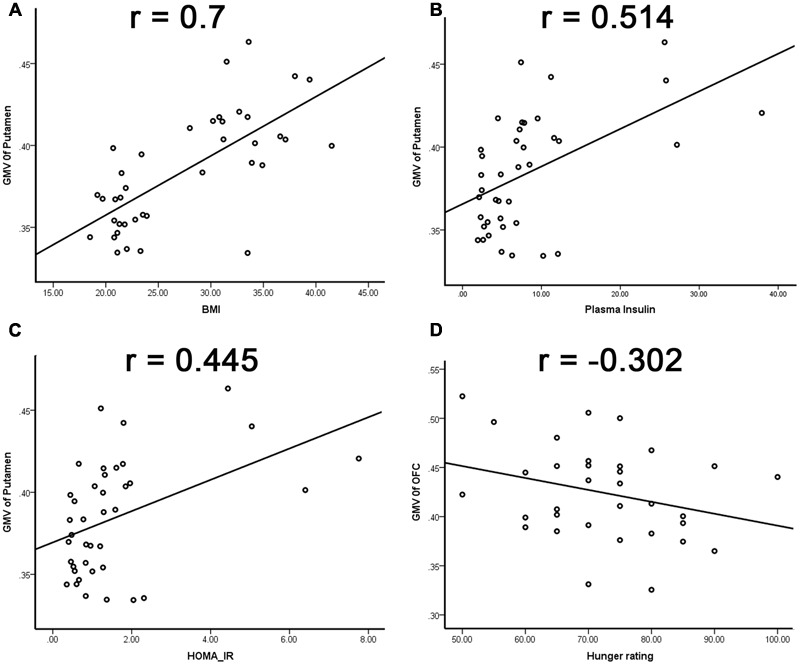
**Relationship between region GMV and physiological factors. (A)** Positive correlation between GMV of the left putamen and BMI; **(B)** positive correlation between GMV of the left putamen and plasma insulin; **(C)** positive correlation between GMV of the left putamen and HOMA-IR; **(D)** negative correlation between the GMV of OFC and hunger rating.

The GMV of the left putamen was positively correlated with BMI (*r* = 0.7, *p* < 0.001; **Figure 2A, Table 2**), plasma insulin (*r* = 0.514, *p* = 0.001; **Figure 2B, Table 2**), and HOMA-IR (*r* = 0.445, *p* = 0.004; **Figure 2C, Table 2**), and the GMV of the OFC was negatively correlated with hunger rating (*r* = -0.302, *p* = 0.047; **Figure 2D, Table 2**).

**Table 2:** Correlation coefficients among all the study variables.

	GMV of left putamen		GMV of OFC	
	*r*	*P*	*r*	*p*
Plasma Insulin	**0.541**	**0.001**	0.242	0.069
HOMA_IR	**0.445**	**0.004**	0.255	0.059
BMI	**0.7**	**0.000**	0.112	0.248
Hunger Rating	-0.161	0.185	**-0.302**	**0.047**

*Bolded values mean significant correlation.*

## Discussion

Previous studies showed that altered eating behaviors in obesity is related to the hyper-functioning striatum and hypo-functioning inhibitory (He et al., 2014a,b). In the present study, we would like to investigate whether the structure of these systems altered in obese individuals and the relationship of the regional GMV with plasma insulin. We found obese men showed a significantly increased GMV in the left putamen, which was positively correlated with plasma insulin. But, we did not find the significant differences of GMV in OFC. However, the GMV of the OFC was negatively correlated with hunger rating. Our findings contribute to the existing literature by reporting regional deviation, altered structure of striatum system in obese individuals.

The current study found that obese subjects showed significantly increased GMV in the left putamen. Previous studies have shown that the putamen is a core region of the impulsive system (Bechara, 2005; Noel et al., 2013), and it plays an important role in goal-directed control of behavior in motivational contexts (Jimura et al., 2010). The study of Rothemund et al. (2007) showed that BMI could predict activation of the putamen during high caloric food viewing. Furthermore, our previous study showed that obese men had increased activity in the left putamen during hunger state (Zhang et al., 2015b). In all, our functional MRI findings and previous research supported that a hyper-functioning striatum exists in obesity; additionally, the current study showed an abnormal structure of the putamen, which may be the reason for excess food intake in obese individuals.

In the current study, there was no difference in plasma glucose between the two groups. However, the levels of plasma insulin and HOMA-IR in obese men were greater than in HCs; in particular, the mean value of HOMA-IR in the obese group was three times more than that in the HCs, which indicated that the islet function of obese subjects in our study was altered, namely due to insulin resistance (Wallace et al., 2004; Chiu et al., 2007). Our findings were consistent with previous studies showing that insulin resistance was associated with obesity (Boden et al., 2005; Isganaitis and Lustig, 2005) and that the pathological condition would progress to Type 2 diabetes. Meanwhile, the GMV of the left putamen was positively correlated with BMI and HOMA-IR, which indicated that the altered GMV of the left putamen may be an important biomarker of the insulin resistance. And, the current study showed that the GMV of the left putamen was positively correlated with plasma insulin, which might clarify that the putamen is a core region participating in insulin signal regulation. However, it is still unclear how the putamen regulates the insulin signal, which need the future research to investigate.

The prefrontal region plays an important role in decision-making and inhibitory control, and several researchers have shown that the PFC regulates the cognitive control of food intake (Grabenhorst et al., 2008; Davis et al., 2010). Previous studies have found increased activation of the OFC in response to high calorie food images (Killgore and Yurgelun-Todd, 2005; He et al., 2014a), and obese individuals showed less activation of the PFC when attempting to inhibit responses to food images, compared to lean subjects (Batterink et al., 2010). Furthermore, our previous study showed that the obese male individuals had significantly decreased neural activity in the OFC during hunger state (Zhang et al., 2015b). Abnormal eating behavior in obesity is related to hypo-functioning inhibitory control, and the OFC is the key region of the inhibitory system (He et al., 2014a,b). In the current study, we did not find a significant difference between obese and healthy subjects in the GMV of the OFC; however, the GMV of the OFC was negatively corrected with hunger rating. Accordingly, the GMV of the OFC could be related more directly to increased subjective motivation toward increased eating behavior.

The current study had several limitations to be addressed in the future. First, we only enrolled male subjects in the current study. Previous studies have shown that obesity in males and females follows different routes, which can affect insulin resistance (Aldhoon-Hainerova et al., 2014; Bosch et al., 2015). With the moderate sample size, it would have become problematic to investigate potential interactions. We will enlarge the sample size and involve subjects of both genders in a future study. Second, we did not assess cognitive and affective status, which are important factors associated with obesity. In the future, we will perform some psychological testing and diagnostic interviews for mental disorders.

## Conclusion

Our study showed an abnormal GMV of the left putamen, which was positively correlated with BMI, plasma insula and HOMA-IR, so the putamen could be a core region participating in insulin signal regulation, and an abnormal structure and function of the putamen could play important roles in obesity and aberrant insulin. Additionally, the current study showed that the GMV of the OFC was negatively corrected with the hunger rating, which indicated that the OFC could be related more directly to increased subjective motivation toward increased eating behavior. The putamen and OFC are two key regions associated with obesity and eating behavior, and structural abnormalities of the two regions might contribute to causing obesity.

## Ethics Statement

This study was carried out in accordance with the recommendations of International Committee of Medical Journal Editors with written informed consent from all subjects. All subjects gave written informed consent in accordance with the Declaration of Helsinki. The protocol was approved by the institutional review board of Tianjin Medical University General Hospital.

## Author Contributions

BZ, CY, and DT designed the work. BZ, XT, CY, QW, JHW, and DT did acquisition and analysis. BZ, XT, CL, and JJW interpreted of data. BZ, XT, CY, JHW, DT, and CL wrote the draft. BZ, XT, CY, JJW, JHW, CL, QW, and DT approved the final version. All authors reviewed the manuscript.

## Conflict of Interest Statement

The authors declare that the research was conducted in the absence of any commercial or financial relationships that could be construed as a potential conflict of interest.

## Footnotes

1 www.fil.ion.ucl.ac.uk/spm

2 http://fmri.wfubmc.edu/
